# A dual domain systematic review and meta-analysis of risk tool accuracy to predict cardiovascular morbidity in prehypertension and diabetic morbidity in prediabetes

**DOI:** 10.3389/fendo.2025.1527092

**Published:** 2025-07-22

**Authors:** William J. Waldock, Nicholas Tekkis, Joe Zhang, Hutan Ashrafian

**Affiliations:** ^1^ Imperial Healthcare UK National Health Service (NHS) Trust, London, United Kingdom; ^2^ Institute of Global Health Innovation, Imperial College London, London, United Kingdom

**Keywords:** prehypertension, prediabetes, diabetic morbidity, cardiovascular disease, screening

## Abstract

**Objective:**

Health forecasting predicts population trends through risk prediction algorithms which can estimate the risk of future disease developing. Screening algorithms can systematically identify patients with a high probability of undiagnosed diseases for diagnostic testing. We describe a dual domain systematic review and meta-analysis of the accuracy of available risk tools to (1) predict prehypertensive deterioration to cardiovascular morbidity, & (2) predict prediabetes deterioration to diabetic morbidity.

**Materials and Methods:**

The primary outcome was the accuracy of the risk scores, and the secondary outcomes were the reporting quality and risk of bias. The dual domain systematic review included studies involving risk tools for (1) prehypertensive adults to predict cardiovascular morbidity (including hypertension, stroke and coronary heart disease) and (2) prediabetic adults to predict diabetic morbidity (including Type 2 Diabetes and end organ damage, such as diabetic nephropathy). Following PROSPERO registration (IDs 425686 & 425683), searches were conducted in PubMed, MEDLINE and Google Scholar.

**Results:**

Accuracy of risk prediction in prehypertension and prediabetes was high: the pooled C statistic for All Cause Cardiovascular Disease was 0.77 (CI 0.71, 0.84) and the pooled Sensitivity for All Cause Diabetic Disease Spectrum risk was 0.68 (CI 0.65, 0.7). However, we found high risk of bias, with inconsistent reporting in both prehypertension and prediabetes papers.

**Discussion:**

We propose nine recommendations for policymakers and commissioners, organised under an “A to I” framework.

**Conclusion:**

We found that predictive performance was generally accurate. However, there remain limitations due to methodological inconsistency, such as timeframe, which undermines comparison.

## 1 Introduction

The chronic disease burden on health systems is a global challenge. Half of the US population has a chronic disease, and 86% of health costs are attributable to chronic disease (1). Health systems are struggling to plan resource distribution to respond. There are two components of the necessary solution, health forecasting and predisease screening. Health forecasting predicts trends in future health events at a population level. This is achieved through risk prediction algorithms which can estimate the risk of future disease developing. Screening algorithms can systematically identify patients with a high probability of undiagnosed diseases for diagnostic testing. Predisease is of particular interest as a precursor of chronic morbidity.

Accurate health forecasts enable improvements in preventive health services, generate patient flow alerts and reduce staff costs. Prehypertension is defined as a systolic blood pressure of 120-139mmHg, and a diastolic blood pressure 80-89mmHg (2), and is a precursor to cardiovascular disease, such as stroke and myocardial infarction. Moreover, in the UK, one in four adults suffer from high blood pressure, it is the third most common reason for premature death, at least half of heart attacks and strokes are associated with hypertension, and it can lead to chronic organ failure and premature death (3). Prediabetes is a non-diabetic hyperglycaemic state (4) which enables warning of the development of diabetic disease; in the UK, around 7 million people are estimated to have prediabetes and therefore have a high risk for developing type 2 diabetes (5). Understanding potential trajectories in health directs long-term investments and policy implementation. This warning of chronic disease makes prediabetes and prehypertension amongst the most impactful targets of risk model products.

Past work on forecasting has provided an incomplete landscape of future health scenarios, highlighting the need for a more robust modelling platform to inform policy (6). In-home care which delivers intervention preemptively may reduce costs associated with non-urgent hospital care (7), and thus allow health forecasting to inform the allocation of resources. Through embedding risk scores into digital health tools, prediction capabilities can help patient self-care and doctor management plans. An electronic personal health record is one type of technology commonly used to support diabetes self-management (8). Preemptive analysis of electronic health records (EHRs) is vital for patient safety. The use of digital health tools could save approximately $7 billion a year in U.S. healthcare spending, equivalent to 1.4% of total expenditures (9). If artificial intelligence can assist in the accurate identification of groups in a population most at risk of developing chronic disease, resource allocation will be more effective. In the UK, ‘Core20Plus5’ (10) is an initiative to reduce healthcare inequalities, in which a target outpatient population of the most deprived 20% of society and five key diagnostic priorities (including hypertension and lipid management) are prioritised, simultaneously saving resources and improving health engagement.

The deterioration of model performance due to drift and bias present two major governance challenges to global health policy leaders. Whilst artificial intelligence may assist in addressing the priorities of ‘Core20Plus5’, there are risks that alternative inequalities may be exacerbated by model bias. For example, hypertension disproportionately affects Afro-Caribbean ethnicities (11); in 2019, an algorithm built using historical data reportedly produced healthcare predictions that favoured white people above black people in the US (12). Nevertheless, this project is directly in line with the official objectives of the Commonwealth Fund, the WHO and UK National Health Policy, and will support the UK’s digital transformation (13); it will act on the ES(H)G investment principles set out in the Business for Health initiative (14) and supports the ambitions of Our Future Health (15). Herein, we describe a dual domain systematic review and meta-analysis of the accuracy of available risk tools to predict prehypertensive deterioration to cardiovascular morbidity & prediabetes deterioration to diabetic morbidity.

## 2 Methods

This dual domain systematic review and meta-analysis was conducted according to a registered protocol and is reported according to the Preferred Reporting Items for Systematic Reviews and Meta-Analyses (PRISMA) statement (16).

### 2.1 Information sources and search strategy

Following the PROSPERO registration (IDs 425686 & 425683), a systematic literature search was performed across multiple databases to identify relevant studies for reviews on Prediabetes and Prehypertension. Initial searches were conducted in PubMed, MEDLINE, and Google Scholar. Secondary searches in EMBASE, The Cochrane Library, Health Technology Assessment Database, and Web of Science yielded only duplicate records, which were removed during the deduplication process in Covidence. Covidence was also used for abstract screening and to manage references throughout the review process. The search strategy was structured with both keyword and MeSH terms to ensure comprehensive coverage of relevant literature. The full systematic search included all publications available up to 10/05/2023. For each review, we specified MeSH terms alongside keywords to target specific populations, conditions, and risk assessments:

Prediabetes Review

Keywords: “diabetes AND risk tool AND prediabetes” OR “diabetes AND risk score AND prediabetes.”

MeSH Terms:

“Diabetes Mellitus, Type 2”

“Prediabetic State”

“Risk Assessment”

“Risk Factors”

Prehypertension Review

Keywords: “risk tool AND prehypertension AND cardiovascular disease” OR “risk score AND prehypertension AND cardiovascular disease.”

MeSH Terms:

“Hypertension”

“Prehypertension”

“Cardiovascular Diseases”

“Risk Assessment”

“Risk Factors”

Search Parameters and Filters

Boolean operators (AND, OR) were employed to refine and combine search terms effectively. Searches were limited to studies published in English and involving human participants, with no restrictions on publication date. In the final stage, advice was sought from the library services at Imperial College London to further refine the search protocol.

Data Management

Search results from all databases were uploaded into Covidence, which was used to remove duplicates, manage citations, and streamline the abstract screening process.

### 2.2 Eligibility criteria

The exclusion criteria were if the article was not in English, and not about the (1) prehypertension to hypertension, or (2) prediabetes to diabetes disease spectrum respectively, not reporting accuracy data, not a prediction tool, the subjects included children aged (0-17), or a meta-analysis, Editorial/Opinion Article.

### 2.3 Selection process

The selection process was performed in three stages: first, titles were screened for relevance. Second, abstracts of the selected titles were reviewed. Finally, full-text articles were assessed for eligibility. Duplicates were removed using Covidence software, and all stages were performed independently by two reviewers (WW & NT). Any discrepancies were resolved by consulting a third reviewer (HA).

### 2.4 Risk of bias

Two review authors (WW & NT) independently screen assessed the risk of bias with the prediction model risk of bias assessment tool PROBAST, which is organised into the following 4 domains: participants, predictors, outcome, and analysis (17). This explores how weaknesses in study design, conduct, or analysis can lead to systematically distorted estimates of model predictive performance (17). Any discrepancy involved a third senior supervisor colleague (HA) being consulted.

### 2.5 Data extraction

Two independent review authors assisted in the stat extraction and subsequent meta-analysis. It was recorded in a mutually shared Excel file with two researchers checking the results. Any discrepancy involved a third colleague being consulted. Individual studies which met the inclusion criteria were included in the statistical analysis, with checks included to ensure no duplication of results under analysis. In the event of an apparent duplication, analysis only included new data from additional studies not already represented.

### 2.6 Data synthesis

These search strategies were kept separate. The dual domain systematic review to concomitantly appraise two risk tools included studies involving risk tools for (1) prehypertensive adults to predict cardiovascular morbidity (including hypertension, stroke and coronary heart disease) and (2) prediabetic adults to predict diabetic morbidity (including Type 2 Diabetes and end organ damage, such as diabetic nephropathy). The dual domain systematic review was conducted in Covidence with data extracted for analysis according to the following categories: study, author, year, population, risk score, disease, time period and accuracy. It was recorded in a mutually shared Excel file with two researchers (WW & NT) checking the results.

Risk ratios for individual studies were combined using a random-effects meta-analysis, which presents the extent of between-study variation and enables Chi^2^, I^2^ & Tau^2^ heterogeneity analysis. Only studies predicting cardiovascular disease or diabetic disease, respectively, over a fixed time period were considered. The different risk tools and their respective performance in predicting cardiovascular & diabetic morbidity were analysed as subgroups. The software used to conduct the meta-analysis was StataCorp. ((2017). Stata Statistical Software: Release 15. College Station, TX: StataCorp LLC). We provided a narrative synthesis of the study findings and meta-analysis of the accuracy of the two domains of predisease risk tools.

## 3 Results

### 3.1 Study selection

#### 3.1.1 Prehypertension

**Figure 1:**
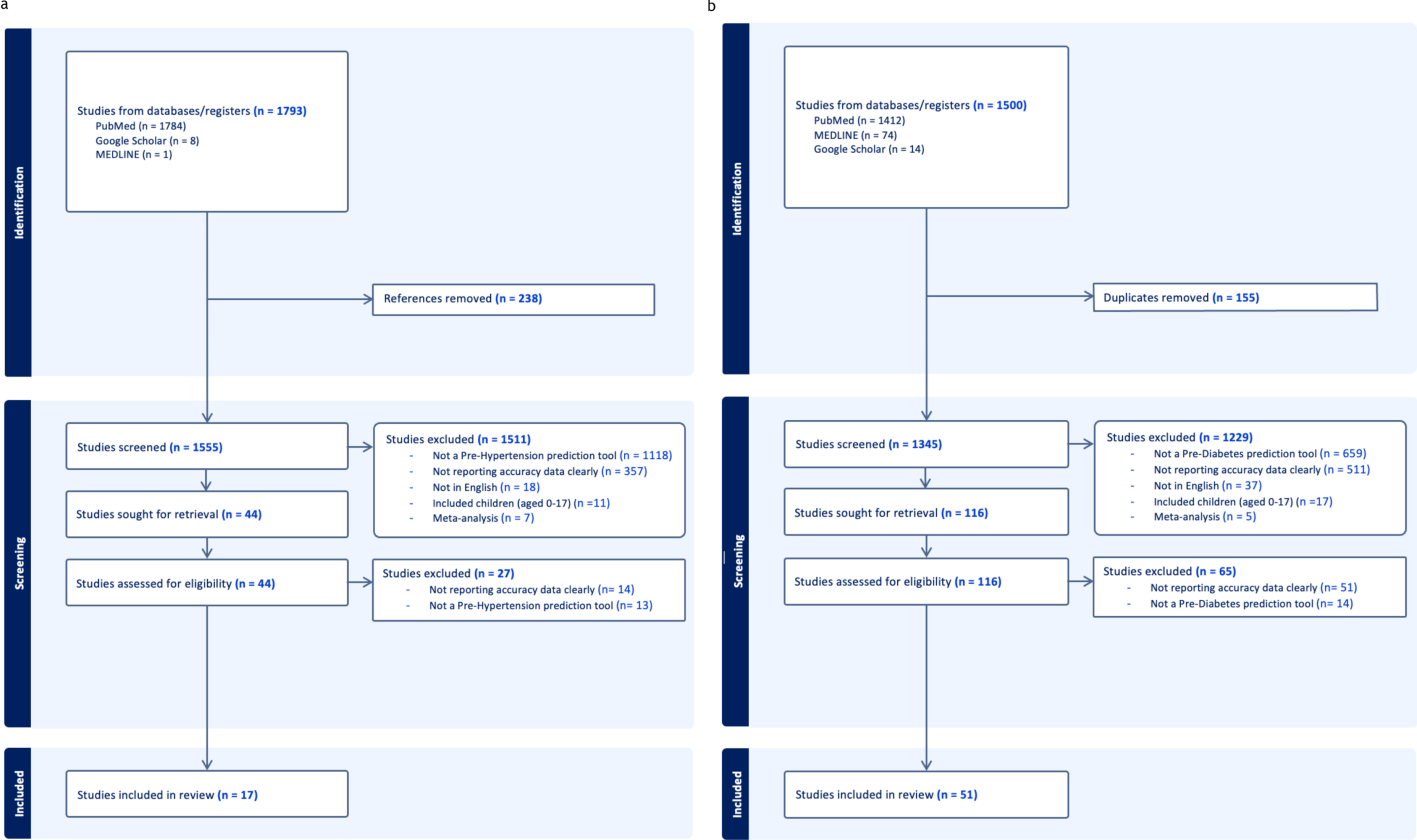
**(a)** Prehypertension PRISMA Diagram. **(b)** Prediabetes PRISMA diagram.

**Figure 2:**
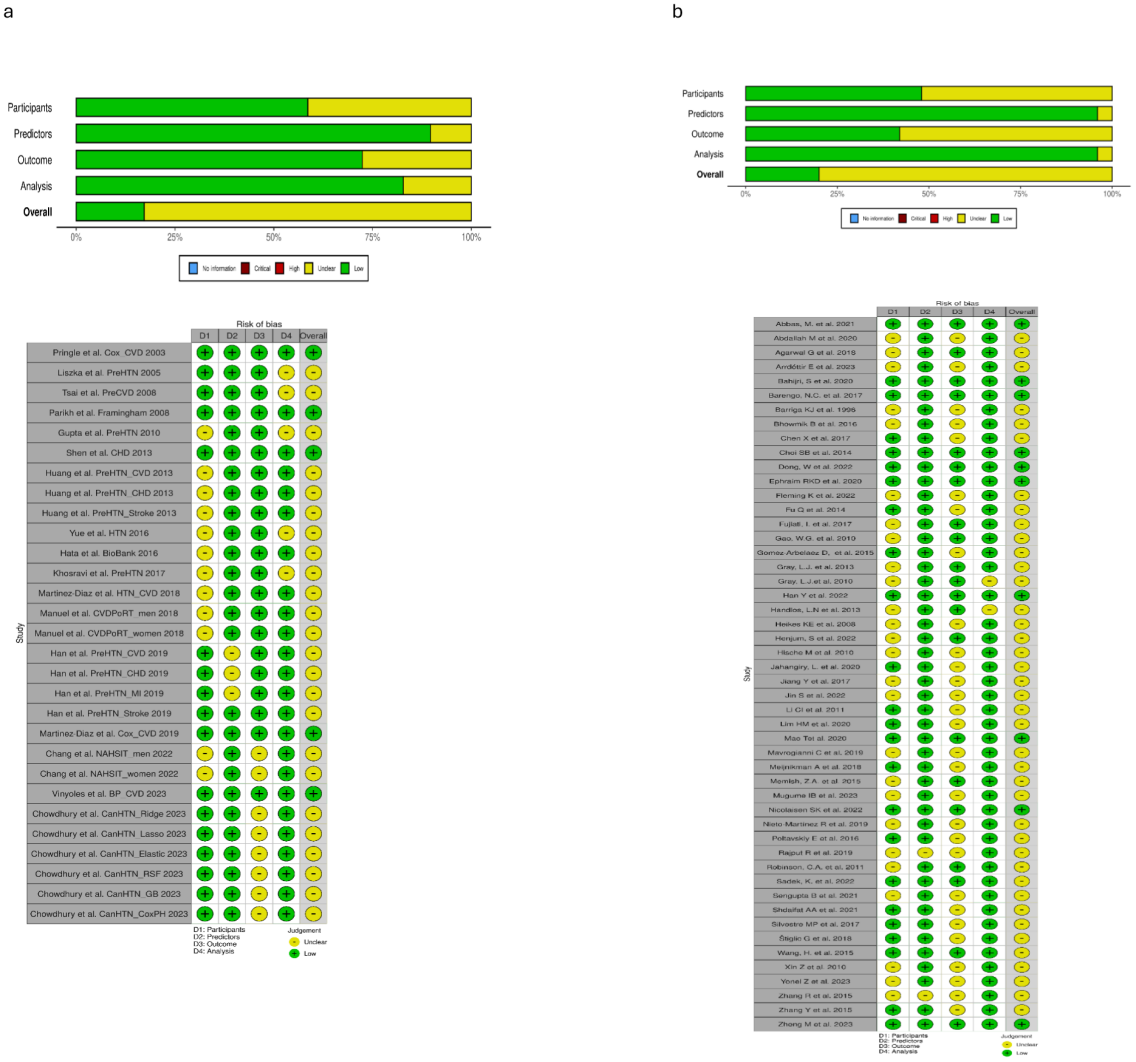
**(a)** Prehypertension risk of bias PROBAST (17) diagrams. **(b)** Prediabetes risk of bias PROBAST (17) diagrams.

**Figure 3:**
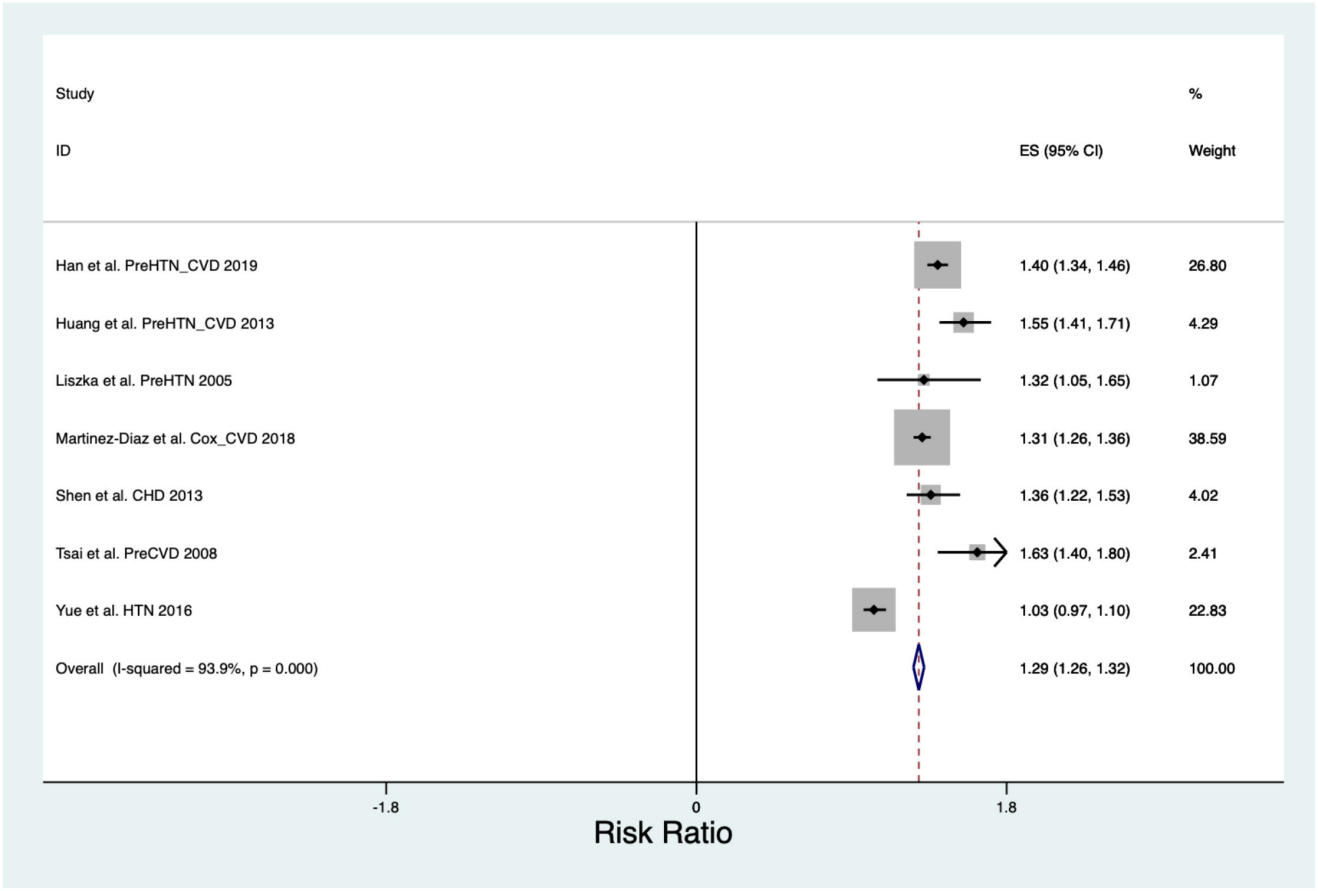
All cause cardiovascular disease risk ratio forest plot.

**Figure 4:**
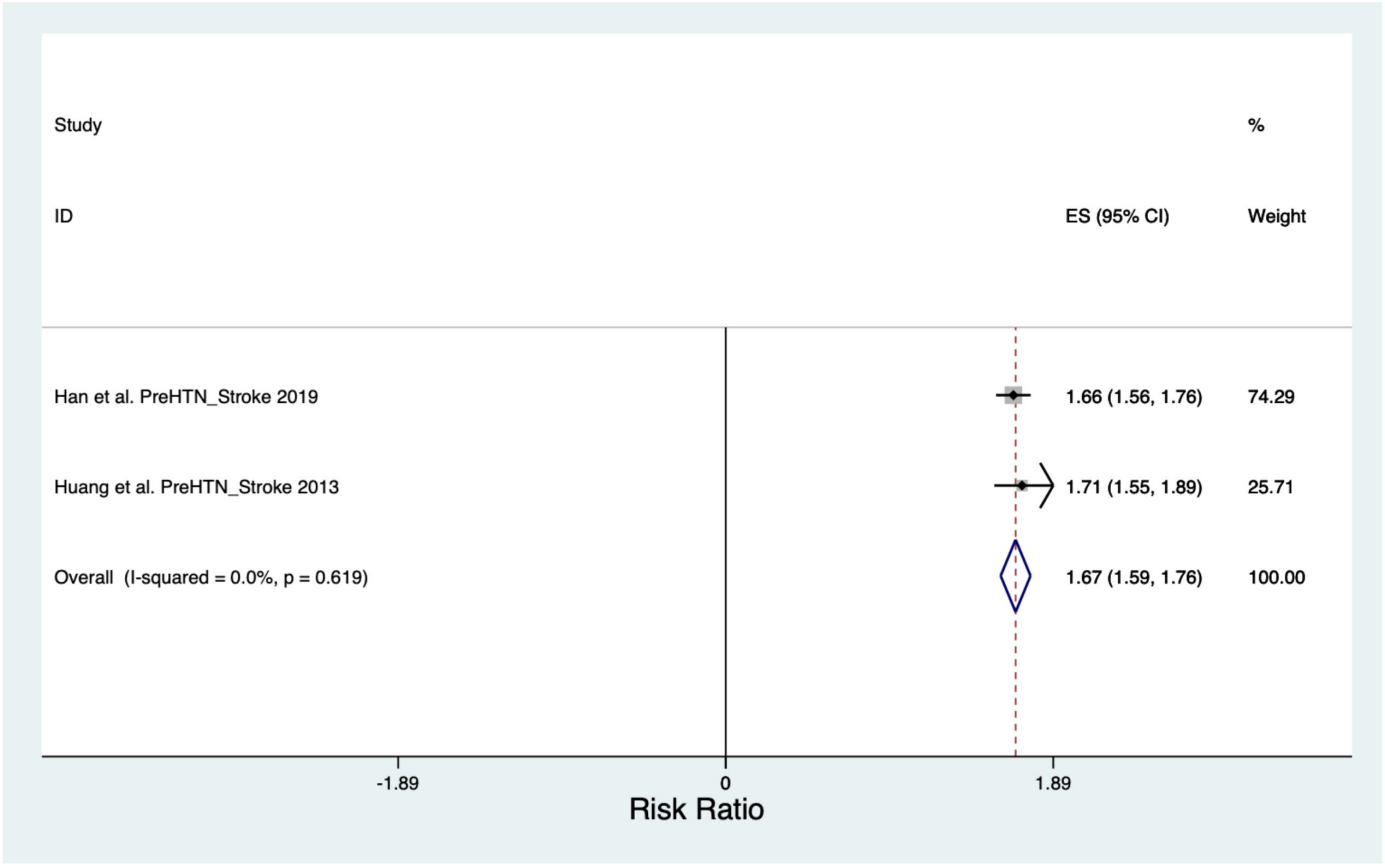
Stroke risk ratio forest plot.

The prehypertension search identified 1793 relevant citations. After removing duplicate results, 1555 articles were screened for titles and abstracts, and 44 studies were included for full-text review. 27 articles were excluded after full-length review due to lack of predictive clarity as per the PROBAST criteria. Thus,17 studies were eligible for inclusion in the study (**Figure 1a**), with a total of 3,077,131 patients represented in the final meta-analysis, after accounting for the risk of double counting patients in different studies. The number of patients involved in each study ranged from 302 to 1,129,098, and the descriptive variables are displayed in **Table 1a**. **Table 2a** provides a Summary of Results. **Figure 2a** describes the PROBAST (17) Risk of Bias assessment. **Figures 3** and **4** describe the subgroups of results.

**Table 1a:** Prehypertension descriptive variables table.

Citation	Subgroup	Participants	n (population)	Modelling method	Geography
(18)	Pringle et al. Cox_CVD 2003	744 participants (elderly hypertensive European population)	744	Systolic blood pressure variability Cox regression model	Europe
(19)	Liszka et al. PreHTN 2005	Analyses were conducted on participants in the National Health and Nutrition Examination Survey I (1971-1975) observed for 18 years for major cardiovascular disease events.	32,000	Cox proportional hazard ratios were calculated to assess stroke, myocardial infarction, and heart failure, in participants with prehypertension and normal blood pressure (<120/80 mm Hg).	USA
(20)	Tsai et al. PreCVD 2008	The cohort consisted of 35,259 adults (>==40 years) with a medium follow-up of 15 years.	35,259	These predisease risk factors included prediabetes, prehypertension, overweight and borderline hypertriglycerdemia and were defined as: fasting glucose at 110–125 mg/dL, systolic blood pressure at 120–139 mmHg, body mass index at 25-29.9 kg/m(2) and serum triglyceride at 150–199 mg/dL, respectively.	USA
(21)	Parikh et al. Framingham 2008	1717 nonhypertensive white individuals 20 to 69 years of age (mean age, 42 years; 54% women)	1,717	Scores were developed for predicting the 1-, 2-, and 4-year risk for new-onset hypertension, and performance characteristics of the prediction algorithm were assessed by using calibration and discrimination measures.	USA
(22)	Gupta et al. PreHTN 2010	Clinically healthy disease-free adults with prehypertension (PreHTN: BP120–139/80–89 mm Hg) have an adverse cardiometabolic risk profile. A statistical analysis of disease-free adult NHANES participants was conducted from 1999 to 2006.	41,474	A statistical analysis of disease-free adult NHANES participants was conducted from 1999 to 2006. Overall prevalence of PreHTN in disease-free adults was 36.3%. Prevalence was higher in men (P<0.001) increasing with age up to 70 years (P<0.001).	USA
(23)	Shen et al. CHD 2013	A systematic search of published research was conducted through January 2013, using electronic databases and bibliographies of retrieved reports.	934,106	Studies were included if they reported multivariate-adjusted relative risks (RRs) and corresponding 95% confidence intervals (CIs) of CHD with respect to prehypertension. A random-effects model was used to combine the study-specific risk estimates.	China
(24)	Huang et al. PreHTN_CVD 2013	Databases (PubMed, EMBASE and the Cochrane Library) and conference proceedings were searched for prospective cohort studies with data on prehypertension and cardiovascular morbidity. Two independent reviewers assessed the reports and extracted data.	468,561	The relative risks (RRs) of CVD, coronary heart disease (CHD) and stroke morbidity were calculated and reported with 95% confidence intervals (95% CIs). Subgroup analyses were conducted on blood pressure, age, gender, ethnicity, follow-up duration, number of participants and study quality.	China
	Huang et al. PreHTN_CHD 2013		292,026		
	Huang et al. PreHTN_Stroke 2013		406,539		
(25)	Hata et al. BioBank 2016	BioBank Japan database, 15,058 patients aged ≥40 years with chronic ischemic CVD (ischemic stroke or myocardial infarction) were divided randomly into a derivation cohort (n = 10,039) and validation cohort (n = 5019).	15,058	Risk prediction models for all-cause and cardiovascular death were developed using the derivation cohort by Cox proportional hazards regression (age, sex, CVD subtype, hypertension, diabetes, total cholesterol, body mass index, current smoking, current drinking, and physical activity)	Japan
(26)	Yue et al. HTN 2016	A prospective cohort study of relative risks and 95% CIs about the comparison with ideal blood pressure, the prehypertension and the all-cause mortality or the death of cardiovascular that corrected a variety of risk factors.	1,129,098	By reading the rest of the 50 in full text, 30 documents were further excluded which included 13 ones that were not compared with the relative risk of prehypertension and ideal blood pressure, 11 ones did not report the RRs and 95% CIs.	China
(27)	Khosravi et al. PreHTN 2017	Iranian population aged 35 years and older, Isfahan Province; Of the 6323 subjects scheduled for assessment of diabetes state 617 were diabetics and 712 were prediabetic.	2,500	COX regression test analysing only prehypertension, prediabetes and its combination and adjusted for gender and age variables	Iran
(28)	Martinez-Diaz et al. HTN_CVD 2018	302 hypertensive patients hospitalized between 2015 and 2017 in Spain.	302	The main variable was time-to-death (all-cause mortality). Secondary variables (potential predictors of the model) were: age, gender, smoking, blood pressure, Charlson Comorbidity Index (CCI), physical activity, diet and quality of life	Spain
(29)	Manuel et al. CVDPoRT_men 2018	104–219 respondents aged 20 to 105 years. There were 3709 cardiovascular events and 818–478 person-years follow-up in the combined derivation and validation cohorts.	12,167	Predictors included body mass index, hypertension, diabetes, and multiple behavioural, demographic and general health risk factors.	Canada
	Manuel et al. CVDPoRT_women 2018		14,801		
(30)	Han et al. PreHTN_CVD 2019	PubMed, Embase, and Web of Science were searched for articles published up to 7 November 2018. Normal range BP was considered SBP less than 120 mmHg and DBP less than 80 mmHg.	491,666	Prehypertension, particularly high-range, is associated with increased risk of total CVDs, CHD, MI, and stroke. Effective control of prehypertension could prevent more than 10% of CVD cases.	China
	Han et al. PreHTN_CHD 2019		256,766		
	Han et al. PreHTN_MI 2019		86,513		
	Han et al. PreHTN_Stroke 2019		445,863		
(31)	Martinez-Diaz et al. Cox_CVD 2019	303 hypertensive patients admitted through the Emergency Department in a Spanish region	303	Cox Regression predictors in the points system were: gender, age, myocardial infarction, heart failure, peripheral arterial disease and daily activity (quality of life).	Spain
(32)	Chang et al. NAHSIT_men 2022	The model was built using the Nutrition and Health Survey in Taiwan (NAHSIT) collected from 1993–1996 and linked with 10 years of events from NHI data.	1,658	The Taiwanese Survey on Hypertension, Hyperglycaemia, and Hyperlipidemia (TwSHHH), conducted in 2002 was used for external validation. The NAHSIT data consisted of 1658 men and 1652 women aged 35–70 years.	Taiwan
	Chang et al. NAHSIT_women 2022		1,652		
(33)	Vinyoles et al. BP_CVD 2023	3,907 subjects (All patients over 18 years of age, without cardiovascular disease, with a first valid 24-hour ABPM and a complete baseline visit carried out in the period between 2009–2014 in Catalonia, were included.)	3,907	Ambulatory Blood Pressure	Spain
(34)	Chowdhury et al. CanHTN_Ridge 2023	18,322 participants on 24 candidate features from the large Alberta’s Tomorrow Project (ATP), aged 35–69 years.	18,322	Penalized regression Ridge model	Canada
	Chowdhury et al. CanHTN_Lasso 2023			Lasso model	
	Chowdhury et al. CanHTN_Elastic 2023			Elastic Net (EN) model	
	Chowdhury et al. CanHTN_RSF 2023			Random survival forest (RSF) model	
	Chowdhury et al. CanHTN_GB 2023			Gradient boosting (GB) model	
	Chowdhury et al. CanHTN_CoxPH 2023			Cox PH model	

Subgroups are described with a unique identifier referring to score applied as shown in **Table 1**.

**Table 1b:** Prediabetes descriptive variables table.

Reference	n (Population)	Geography	Modelling method
(35)	486,495	Denmark	2012–2018; prediction model included HbA1c, age, sex, body mass index (BMI), any antihypertensive drug use, pancreatic disease, cancer, self-reported diet, doctor’s advice to lose weight or change dietary habits, having someone to talk to, and self-rated health.
(36)	4,566	Republic of Korea	1 year; two models to screen for prediabetes using an artificial neural network (ANN) and support vector machine (SVM)
(37)	1,546,269	China	January 2006 to December 2017; eXtreme Gradient Boosting (XGBoost), random forest (RF), Logistic Regression (LR), and Fully connected neural network (FCN) as classifiers, four models were constructed to distinguish NFG, IFG and T2DM.
(38)	1,454	Qatar	Same dataset as development data (20/80 split)
(39)	NA	Saudi Arabia	No validation performed; performance data is from model development
(40)	NA	Colombia	No validation performed; performance data is from model development
(41)	619	China	Same dataset as development data (33/66 split)
(42)	6,933	Indonesia	External population-based health survey dataset
(43)	4,336	China	
(44)	3,171	UK	
(45)	1,304	Portugal	External prospective 1-year follow-up data on the city-wide cohort
(46)	2,155	Middle East	Same dataset as development data
(47)	308	Algeira	
(48)	50	Saudi Arabia	
(49)	1,857	Canada	Same dataset as development data (30/70 split)
(50)	930	Qatar	External population-based BioBank dataset
(51)	NA	Slovenia	No validation performed; performance data is from model development
(52)	1,186	China	3 External population-based health survey datasets from different regions of Chi
	3,162		
	1,289		
(53)	713	China	25–64 years were recruited from a Shanghai population from July 2019 to March 2020. Glucose status was tested using haemoglobin A1c (HbAlc), 2h-post-load glucose (2hPG), and fasting blood glucose (FBG). The FINDRISC questionnaire and the metabolic syndrome were examined. The performance of the FINDRISC was assessed using the area under the receiver operating characteristic curve (AUC-ROC).
(54)	220	Finland	1 March 2020 and 15 May 2021; Data collection took place via face-to-face interviews between 1 March 2020 and 15 May 2021. Participation included answering the Finnish Diabetes Risk Score (FINDRISC), measuring the HbA1c levels and background information.
(55)	1,479	Canada	Phase 1 (2007 to 2011) and Phase 2 (2013 to 2014) of the CANRISK study; Sensitivity and specificity of CANRISK scores using published risk score cut-off points were calculated. Logistic regression was conducted with alterative ethnicity-specific BMI and WC cut-off points to predict dysglycaemia using CANRISK variables.
(52)	6,197	China	2006–2007; Performance of the scores was measured with the Hosmer-Lemeshow goodness-of-fit test and ROC c-statistic. Age, waist circumference, body mass index and family history of diabetes were included in the risk score for both men and women, with the addition factor of hypertension for men. The ROC c-statistic was 0.70 for both men and women in the derivation samples.
(56)	406	Australia	May-October 2019; All patients received a point of care (POC) HbA1c test. HbA1c test results were categorised into diabetes (≥6.5% or ≥48 mmol/mol), prediabetes (5.7-6.4% or 39–47 mmol/mol), or normal (<5.7% or 39 mmol/mol).
(57)	1,764	Kenya	April-June 2015; The performance of the FINDRISC tools in predicting undiagnosed diabetes was assessed using the area under the receiver operating curve (AU-ROC). Non-parametric analyses of the AU-ROC, Sensitivity (Se), and Specificity (Sp) of FINDRISC tools were determined.
(58)	135	Ghana	April 2019; The FINDRISC questionnaire was used to gather data from the respective participants. Serum glucose and lipids were estimated with enzymatic techniques, and metabolic syndrome (MetS) screened with the international diabetes federation (IDF) criteria.
(59)	28,251	China	2006–2014 and 2006–2008 to 2015 in rural Deqing, Chi; RS models were constructed with coefficients (β) of Cox regression. Receiver-operating characteristic curves were plotted and the area under the curve (AUC) reflected the discriminating accuracy of an RS model.
(60)	772	Colombia	Between June 1, 2012 and October 31, 2012; A modified version of FINDRISC was completed, and the glycemia values from all the subjects were collected from the hospital’s database. Firstly, a cross-section analysis was performed and then, the subsample of prediabetic participants was followed for diabetes incidence.
(61)	424	New Zealand	All participants who completed the FINDRISC questionnaire during a pre-screening phase with a score of ≥12 were then screened using a 2h oral glucose tolerance test (2h-OGTT) to identify undiagnosed dysglycemia.
(62)	3,886	USA	The sensitivity, specificity, and the receiver operating characteristic (ROC) curve of the testing model were calculated for undiagnosed diabetes and prediabetes, determined by oral glucose tolerance test (OGTT).
(63)	7,675	China	The results showed that the participants with undiagnosed diabetes reported the highest NCDRS value, followed by those with prediabetes (P < 0.001). The best cut-off points of NCDRS for detecting undiagnosed diabetes and prediabetes were 27 (with a sensitivity of 78.0% and a specificity of 57.7%) and 27 (with a sensitivity of 66.0% and a specificity of 62.9%). The AUCs of NCDRS for identifying undiagnosed diabetes and prediabetes were 0.749 (95% CI: 0.739~0.759) and 0.694 (95% CI: 0.683~0.705). These results demonstrate the excellent performance of NCDRS in screening undiagnosed diabetes in the community population in eastern Chi and further provide evidence for using NCDRS in detecting prediabetes.
(64)	18,384	China	During a median follow-up of 7.55 years, 5697 new-onset T2D cases were identified. Predictor variables included age, body mass index, waist circumference, diastolic blood pressure, triglycerides, fasting plasma glucose, and fatty liver. The proposed models outperformed five existing models. In internal validation, the AUCs of the coefficient-based models were 0.741 (95% CI 0.723-0.760) for men and 0.762 (95% CI 0.720-0.802) for women. External validation yielded comparable prediction performance.
(65)	293	Malaysia	The prevalence of undiagnosed diabetes was 7.5% and prediabetes was 32.8%. The ROC-AUC of FINDRISC was 0.76 (undiagnosed diabetes) and 0.79 (dysglycaemia). There was no statistical difference between FINDRISC and ModAsian FINDRISC. The recommended optimal FINDRISC cut-off point for undiagnosed diabetes was ≥11 (Sensitivity 86.4%, Specificity 48.7%). FINDRISC ≥11 point has higher sensitivity compared to USPSTF criteria (72.7%) and higher specificity compared to the ADA (9.6%).
(66)	651	Belgium	Of 651 subjects, 50.4% were diagnosed with prediabetes, whereas 11.1% was diagnosed with T2DM. FINDRISC score increased with worsening of glucose status 11 ± 3, 13 ± 4 and 15 ± 5 in respectively, subjects without T2DM, prediabetes and T2DM. 312 subjects had the MetS. The aROC of the FINDRISC to identify subjects with T2DM was 0.76 (95% CI 0.72–0.82), sensitivity was 64% and specificity was 63% with 13 as cutoff point. Adding FPG or HbA1c to FINDRISC, the aROC increased significantly to 0.91(95% CI 0.88–0.95) and 0.93(95% CI 0.90–0.97), respectively (p < 0.001). The aROC of the MetS to identify subjects with diabetes was 0.72 (95% CI 0.65–0.78), sensitivity was 75% and specificity was 55%. The aROC of the FINDRISC + HbA1c was significantly higher than the MetS for predicting T2DM (p < 0.001).
(67)	1,021	Taiwan	The AUCs and their 95% confidence intervals (CIs) were 0.60 (0.54-0.66) for men and 0.72 (0.66-0.77) for women in model 1; 0.62 (0.56-0.68) for men and 0.74 (0.68-0.80) for women in model 2; and 0.64 (0.58-0.71) for men and 0.75 (0.69-0.80) for women in model 3. The AUCs of these three models were all above 0.7 in women, but not in men. No significant difference in either women or men (p = 0.268 and 0.156, respectively) was observed in the AUC of these three models. Compared to 16 tools published in the literature, ADART had the second largest AUC in both men and women.
(68)	440	Iran	A total of 440 adults ages 30–65 years (Mage = 48.8 years, SDage = 11.2 years) were included in the study. Around half of the participants were women (50%), illiterate (51.4%), and married (85.2). In the prediabetes diagnosis scale, the present cut-point yielded a sensitivity of 98.7 (95% CI:96.6–99.6), specificity of 53.1 (95% CI: 44.6–61.5), positive predictive value (PPV) of 81.4 (95% CI:77–85.3), positive predictive value (NPV) of 95.0 (95% CI:87.7–98.6), and accuracy of 83.9 (95% CI:81.4–89.2) with an area under curve (AUC) of 0.84 (95% CI: 0.80 − 0.89).
(69)	1,455	China	Two risk score models for screening postprandial hyperglycemia were developed. The simple model used non-invasive risk factors (age, height, weight, waist, systolic blood pressure, pulse, hypertension, dyslipidaemia and family history of diabetes mellitus), and the full model contained additional variables [fasting blood glucose (FBG), triglyceride/high density lipoprotein cholesterol] obtainable by invasive laboratory tests. The area under receiver operating characteristic curve (AUC) of simple model was similar to FBG and glycated haemoglobin. The full model has the largest AUC [0.799 (0.789-0.809) and 0.730 (0.702-0.758)] in both derivation and validation cohorts (p < 0.001 compared with simple model, FBG, and glycated haemoglobin). At a cutoff point of 80, the sensitivity, specificity and percentage that needed subsequent OGTT were 75.97, 67.56 and 48.38%, respectively.
(51)	2,073	Slovenia	The fil model contained five questions for undiagnosed Type 2 diabetes prediction and achieved an area under the receiver-operating characteristic curve of 0.851 (95% CI 0.850-0.853). The impaired fasting glucose prediction model included six questions and achieved an area under the receiver-operating characteristic curve of 0.840 (95% CI 0.839-0.840). There were four questions that were included in both models (age, sex, waist circumference and blood sugar history), with physical activity selected only for undiagnosed Type 2 diabetes and questions on family history and hypertension drug use selected only for the impaired fasting glucose prediction model.
(70)	9,391	USA	Both scores performed well and robustly, while the ADA score performed somewhat better (e.g., AUC=0.77 for ADA and 0.73-0.74 for CDC for DM; 0.72-0.74 and 0.70-0.71 for preDM). The same predictors and scoring rules seem to be reasonably justified with different cut points for DM and preDM, which can make usage easier and consistent. Some factors such as race and HDL/LDL cholesterols may be useful additions to health education.
(71)	392	Jordan	This study included 392 participants: 231 patients with normal fasting blood sugar (FBG), 101 patients with prediabetes and 60 patients with type 2 diabetes. The FINDRISC, British, and Australian risk scores were strongly inter-correlated and weakly correlated with other systems’ risk scores. Moreover, they correlated moderately and significantly with FBS. In contrast, other systems risk scores were associated weekly with FBS. Based on receiving operating characteristics (ROC) analysis and multivariate logistic regression, the FINDRISC risk score was superior to other risk scores to predict high FBS and identify prediabetes and diabetes.
(72)	303	Canada	A total of 303 individuals participated in the study. Half were aged less than 45 years, two-thirds were female and 84% were Inuit. A total of 18% had prediabetes, and an additional 4% had undiagnosed diabetes. The odds of having dysglycaemia rose exponentially with age, while the relationship with BMI was U-shaped. Compared with lab test results, using a cut-off point of 32 the CANRISK tool achieved a sensitivity of 61%, a specificity of 66%, a positive predictive value of 34% and an accuracy rate of 65%.
(73)	1,351	USA	Fasting glucose, age and body mass index (BMI) were selected as risk variables by CART when simulating the simultaneous approach (SEN = 91%, SPE = 55%).
(74)	NA	USA	The resulting tool, called the Diabetes Risk Calculator, includes questions on age, waist circumference, gestational diabetes, height, race/ethnicity, hypertension, family history, and exercise.
(75)	1,737	Germany	A clinical decision tree included age and systolic blood pressure (sensitivity 89.3%, specificity 37.4%, and positive predictive value (PPV) 48.0%), while a tree based on clinical and laboratory data included fasting glucose and systolic blood pressure (sensitivity 89.7%, specificity 54.6%, and PPV 56.2%). The inclusion of additional parameters did not improve test quality. The external validation approach confirmed the presented decision trees.
(76)	2,261	China	The significant risk factors included in the logistic regression method were age, body mass index, waist/hip ratio (WHR), duration of hypertension, family history of diabetes, and history of hypertension for T2DM and T2DM plus PDM. In the classification tree analysis, WHR and duration of hypertension were the most important determining factors in the T2DM and T2DM plus PDM model.
(77)	3,339	UK	External validation of the model and score employed an independent data set comprising 2,359 participants with 357 events. Predictive performance, discrimination, calibration, and clinical utility were assessed. The fil model included age, sex, body mass index, smoking status, first-degree relative with diabetes, presence of a dental prosthesis, presence of mobile teeth, history of periodontal treatment, and probing pocket depths ≥5 mm as well as prespecified interaction terms.
(78)	2,116	Europe	The AUC-ROC for undiagnosed T2DM was 0.824 with optimal cut-off ≥14 (Se = 68%, Sp = 81.7%) for the total sample, 0.839 with optimal cut-off ≥15 (Se = 83.3%, Sp = 86.9%) for HICs, 0.794 with optimal cut-off ≥12 (Se = 83.3%, Sp = 61.1%) for HICs under austerity measures and 0.882 with optimal cut-off ≥14 (Se = 71.4%, Sp = 87.8%) for LMICs.
(79)	3,454	Venezuela	The prevalence of uT2D and prediabetes were 3.3% and 38.5%. The AUC with the LA-FINDRISC vs. the O-FINDRISC were: for uT2D, 0.722 vs. 0.729 in men (p=0.854) and 0.724 vs. 0.732 in women (p=0.896); for prediabetes (impaired fasting glucose [IFG] + impaired glucose tolerance [IGT], 0.590 vs. 0.587 in men (p=0.887) and 0.621 vs. 0.627 in women (p=0.777); for IFG, 0.582 vs. 0.580 in men (p=0.924) and 0.607 vs. 0.617 in women (p=0.690); for IGT, 0.691 vs. 0.692 in men (p=0.971) and 0.672 vs. 0.671 in women (p=0.974). Using the LA-FINDRISC, the best cut-offs to detect uT2D were 9 in men and 10 in women and to detect IGT was 9 in both genders.
(80)	713	Lebanon	Of 713 subjects, 397 subjects (55.2% female; 44.8% male) completed the blood tests and thus were considered as the sample population. 7.6% had UT2DM, 22.9% prediabetes and 35.8% had MS, where men had higher prevalence than women for these 3 outcomes (P = 0.001, P = 0.003 and P = 0.001) respectively. The AUROC value with 95% Confidence Interval (CI) for detecting UT2DM was 0.795 (0.822 in men and 0.725 in women), 0.621(0.648 in men and 0.59 in women) for prediabetes and 0.710 (0.734 in men and 0.705 in women) for MS. The correspondent optimal cut-off point for UT2DM was 11.5 (sensitivity = 83.3% and specificity = 61.3%), 9.5 for prediabetes (sensitivity = 73.6% and specificity = 43.1%) and 10.5 (sensitivity = 69.7%; specificity = 56.5%) for MS.
(57)	4,027	Kenya	A total of 4,027 data observations of individuals aged 18–69 years were analysed. The proportion/prevalence of undiagnosed diabetes and prediabetes was 1.8% [1.3-2.6], and 2.6% [1.9-3.4] respectively. The AU-ROC of the modified FINDRISC and simplified FINDRISC in detecting undiagnosed diabetes were 0.7481 and 0.7486 respectively, with no statistically significant difference (p = 0.912). With an optimal cut-off ≥ 7, the simplified FINDRISC had a higher positive predictive value (PPV) (7.9%) and diagnostic odds (OR:6.65, 95%CI: 4.43-9.96) of detecting undiagnosed diabetes than the modified FINDRISC.
(41)	619	China	The outcome was defined as a newly detected diabetes mellitus or prediabetes; receiver-operating characteristic curve (AUC-ROC), precision-recall curve (AUC-PR), and calibration plots. Two existing diabetes mellitus risk models were included for comparison.
(81)	325	India	January 1, 2018-December 31, 2019; Fasting blood sugar value was used as the gold standard to validate IDRS. Data were collected using a validated and pretested interview schedule. Data entry and analysis were performed in computer using SPSS-24.
(57)	1,764	Kenya	April and June 2015; Modified FINDRISC
(82)	2,293	Bangladesh	HbA1c
(83)	892	India	PRESS
(84)	619	China	HbA1c

**Table 2a:** Summary results table.

Subgroup	Disease category	C-statistic	Hazard ratio	Risk ratio	Prevalence ratio
Pringle et al. Cox_CVD 2003 (18)	CVD	NA	1.8	NA	NA
Liszka et al. PreHTN 2005 (19)	CVD	NA	NA	1.32	NA
Tsai et al. PreCVD 2008 (20)	CVD	NA	NA	1.63	NA
Parikh et al. Framingham 2008 (21)	HTN	0.788	NA	NA	NA
Gupta et al. PreHTN 2010 (22)	HTN	NA	NA	NA	1.3
Shen et al. CHD 2013 (23)	CVD	NA	NA	1.36	NA
Huang et al. PreHTN_CVD 2013 (24)	CVD	NA	NA	1.55	NA
Huang et al. PreHTN_CHD 2013 (24)	CHD	NA	NA	1.5	NA
Huang et al. PreHTN_Stroke 2013 (24)	Stroke	NA	NA	1.71	NA
Yue et al. HTN 2016 (26)	CVD	NA	NA	1.03	NA
Hata et al. BioBank 2016 (25)	CVD	0.703	1.81	NA	NA
Khosravi et al. PreHTN 2017 (27)	CVD	NA	1.74	NA	NA
Martinez-Diaz et al. HTN_CVD 2018 (28)	CVD	0.76	1.6	NA	NA
Manuel et al. CVDPoRT_men 2018 (29)	CVD	0.82	NA	NA	NA
Manuel et al. CVDPoRT_women 2018 (29)	CVD	0.86	NA	NA	NA
Han et al. PreHTN_CVD 2019 (30)	CVD	NA	NA	1.4	NA
Han et al. PreHTN_CHD 2019 (30)	CHD	NA	NA	1.4	NA
Han et al. PreHTN_MI 2019 (30)	MI	NA	NA	1.86	NA
Han et al. PreHTN_Stroke 2019 (30)	Stroke	NA	NA	1.66	NA
Martinez-Diaz et al. Cox_CVD 2019 (31)	CVD	0.71	NA	1.31	NA
Chang et al. NAHSIT_men 2022 (32)	CVD	0.76	NA	NA	NA
Chang et al. NAHSIT_women 2022 (32)	CVD	0.75	NA	NA	NA
Vinyoles et al. BP_CVD 2023 (33)	CVD	NA	1.49	NA	NA
Chowdhury et al. CanHTN_Ridge 2023 (34)	HTN	0.78	NA	NA	NA
Chowdhury et al. CanHTN_Lasso 2023 (34)	HTN	0.78	NA	NA	NA
Chowdhury et al. CanHTN_Elastic 2023 (34)	HTN	0.78	NA	NA	NA
Chowdhury et al. CanHTN_RSF 2023 (34)	HTN	0.76	NA	NA	NA
Chowdhury et al. CanHTN_GB 2023 (34)	HTN	0.76	NA	NA	NA
Chowdhury et al. CanHTN_CoxPH 2023 (34)	HTN	0.77	NA	NA	NA

(CHD, Coronary Heart Disease; CVD, Cardiovascular disease; MI, Myocardial Infarction; HTN, Hypertension). Subgroups are described with a unique identifier referring to score applied as shown in **Table 1**.

**Table 2b:** Sensitivity, Specificity, PPV, NPV, accuracy and area under the curve of all cause diabetes scores.

Subgroup	n (Population)	Sensitivity	Specificity	PPV	NPV	Accuracy	Area under the curve
Barriga et al. SIM 1996a (73)	583	0.91	0.55	0.31	0.97	NA	0.73
Barriga et al. St1 1996b (73)	768	0.92	0.41	0.26	0.96	NA	0.67
Heikes et al., 2008 (74)	NA	0.75	0.65	0.49	0.85	NA	0.75
Hische et al., 2010 (75)	1,737	0.89	0.37	0.48	NA	NA	NA
Xin et al., 2010 (76)	2,261	0.74	0.72	0.24	0.96	NA	0.73
Gao et al. Men 2010a (43)	1,687	0.86	0.21	NA	NA	NA	0.61
Gao et al. Women 2010b (43)	2,649	0.76	0.44	NA	NA	NA	0.63
Gray et al., 2010a (44)	3,171	0.81	0.45	0.29	0.9	NA	0.72
Li et al. ADART men 2011a (67)	456	NA	NA	NA	NA	NA	0.6
Li et al. ADART women 2011b (67)	565	NA	NA	NA	NA	NA	0.72
Li et al. ADART lifestyle men 2011c (67)	456	NA	NA	NA	NA	NA	0.62
Li et al. ADART lifestyle women 2011d (67)	565	NA	NA	NA	NA	NA	0.74
Li et al. ADART bio men 2011e (67)	456	NA	NA	NA	NA	NA	0.64
Li et al. ADART bio women 2011f (67)	565	NA	NA	NA	NA	NA	0.75
Robinson et al., 2011 (49)	1,857	0.7	0.67	0.35	0.9	NA	0.75
Gray et al. OGTT 2012b	3,004	0.75	0.52	0.29	0.89	NA	0.69
Gray et al. HbA1c 2012c	3,004	0.75	0.5	0.37	0.83	NA	0.67
Gray et al., 2013d (45)	1,304	0.69	0.63	0.38	0.86	NA	0.72
Bhowmik et al. HbA1c >38 PreDB 2013 (82)	2,293	0.68	0.66	0.17	0.96	NA	NA
Bhowmik et al. HbA1c >39 PreDB 2013 (82)	2,293	0.64	0.73	0.18	0.96	NA	NA
Bhowmik et al. HbA1c >42 PreDB 2013 (82)	2,293	0.38	0.89	0.25	0.94	NA	NA
Bhowmik et al. HbA1c >48 PreDB 2013 (82)	2,293	0.15	0.93	0.17	0.92	NA	NA
Bhowmik et al. HbA1c >38 DB 2013 (82)	2,293	0.96	0.69	0.21	0.99	NA	NA
Bhowmik et al. HbA1c >39 DB 2013 (82)	2,293	0.95	0.76	0.25	0.99	NA	NA
Bhowmik et al. HbA1c >42 DB 2013 (82)	2,293	0.86	0.93	0.53	0.99	NA	NA
Bhowmik et al. HbA1c >48 DB 2013 (82)	2,293	0.76	0.98	0.78	0.98	NA	NA
Handlos et al., 2013 (46)	2,155	0.76	0.5	NA	NA	NA	0.7
Choi et al. PreDiab (KNHANES 2010) 2014a (36)	4,566	0.76	0.6	NA	NA	0.63	0.73
Choi et al. PreDiab (KNHANES 2011) 2014b (36)	4,566	0.74	0.56	NA	NA	0.6	0.71
Choi et al. Diab (KNHANES 2010) 2014c (36)	4,566	0.77	0.66	NA	NA	0.67	0.77
Choi et al. Diab (KNHANES 2011) 2014d (36)	4,566	0.74	0.64	NA	NA	0.65	0.75
Fu et al., 2014 (69)	1,455	0.76	0.68	NA	NA	NA	0.8
Memish et al., 2015 (48)	50	0.76	0.68	NA	NA	NA	0.68
Wang et al., 1 Men 2015a (52)	448	NA	NA	NA	NA	NA	0.75
Wang et al., 1 Women 2015b (52)	738	NA	NA	NA	NA	NA	0.77
Wang et al., 2 Men 2015c (52)	898	NA	NA	NA	NA	NA	0.74
Wang et al., 2 Women 2015d (52)	2,264	NA	NA	NA	NA	NA	0.72
Wang et al. 3 Men 2015e (52)	366	NA	NA	NA	NA	NA	0.31
Wang et al. 3 Women 2015f (52)	923	NA	NA	NA	NA	NA	0.5
Wang et al. Men 2015a (52)	2,094	0.57	0.72	0.13	0.96	NA	NA
Wang et al. Women 2015b (52)	4,103	0.69	0.6	0.11	0.96	NA	NA
Gomez-Arbelaez et al. >11 FINDRISC men 2015a (60)	228	0.83	0.49	0.04	0.99	NA	NA
Gomez-Arbelaez et al. >11 FINDRISC women 2015b (60)	544	0.86	0.37	0.04	0.99	NA	NA
Gomez-Arbelaez et al. >12 FINDRISC men 2015c (60)	228	0.67	0.57	0.4	0.98	NA	NA
Gomez-Arbelaez et al. >12 FINDRISC women 2015d (60)	544	0.86	0.45	0.04	0.99	NA	NA
Gomez-Arbelaez et al. >13 FINDRISC men 2015e (60)	228	0.67	0.66	0.05	0.99	NA	NA
Gomez-Arbelaez et al. >13 FINDRISC women 2015f (60)	544	0.79	0.54	0.04	0.99	NA	NA
Gomez-Arbelaez et al. >14 FINDRISC men 2015g (60)	228	0.67	0.75	0.07	0.99	NA	NA
Gomez-Arbelaez et al. >14 FINDRISC women 2015h (60)	544	0.71	0.63	0.05	0.99	NA	NA
Gomez-Arbelaez et al. >15 FINDRISC men 2015i (60)	228	0.5	0.81	0.07	0.98	NA	NA
Gomez-Arbelaez et al. >15 FINDRISC women 2015j (60)	544	0.57	0.71	0.05	0.98	NA	NA
Gomez-Arbelaez et al. >16 FINDRISC men 2015k (60)	228	0.33	0.86	0.06	0.98	NA	NA
Gomez-Arbelaez et al. >16 FINDRISC women 2015l (60)	544	0.5	0.76	0.05	0.98	NA	NA
Gomez-Arbelaez et al. >17 FINDRISC men 2015m (60)	228	0.33	0.88	0.07	0.98	NA	NA
Gomez-Arbelaez et al. >17 FINDRISC women 2015n (60)	544	0.5	0.82	0.07	0.98	NA	NA
Zhang et al. PredD Screening 2015 (62)	3,886	0.74	0.53	NA	NA	NA	NA
Zhang et al. PredD HbA1c 2015 (62)	619	0.61	0.58	0.61	0.57	NA	0.62
Zhang et al. PredD FPG 2015 (62)	619	0.47	0.86	0.78	0.6	NA	0.73
Zhang et al. PredD HbA1c & FPG 2015 (62)	619	0.61	0.77	0.74	0.64	NA	0.75
Zhang et al. DB HbA1c 2015 (62)	619	0.73	0.88	0.69	0.89	NA	0.85
Zhang et al. DB FPG 2015 (62)	619	0.58	0.95	0.8	0.86	NA	0.84
Zhang et al. DB HbA1c & FPG 2015 (62)	619	0.84	0.82	0.64	0.93	NA	0.88
Poltavskiy et al. ADA >4 2016a (70)	9,391	0.78	0.54	0.57	0.76	NA	NA
Poltavskiy et al. >4 2016b (70)	9,391	0.76	0.54	0.53	0.77	NA	NA
Poltavskiy et al. >5 2016c (70)	9,391	0.83	0.57	0.12	0.98	NA	NA
Poltavskiy et al. CDC >9 2016d (70)	9,391	0.74	0.54	0.56	0.73	NA	NA
Poltavskiy et al. >9 2016e (70)	9,391	0.72	0.54	0.51	0.74	NA	NA
Poltavskiy et al. >10 2016f (70)	9,391	0.79	0.5	0.1	0.97	NA	NA
Barengo et al., 2017 (40)	NA	0.57	0.73	0.58	0.76	NA	0.72
Chen et al. T2DM 2017 (59)	28,251	NA	NA	0.02	NA	NA	0.71
Fujiati et al., 2017 (42)	6,933	0.55	0.66	0.12	0.94	NA	0.65
Jiang et al., 2017 (72)	303	0.61	0.66	0.34	NA	0.65	NA
Silvestre et al. FINDRISC 2017 (61)	424	0.6	0.55	NA	NA	NA	0.6
Abraham et al., 2018	651	0.64	0.63	NA	NA	NA	0.76
Stiglic et al., 2018 (51)	2,073	0.73	0.81	0.6	0.89	NA	0.84
Agarwal et al. 33 level 2018a (55)	1,479	0.49	0.8	0.3	0.9	0.76	NA
Agarwal et al., 21 level 2018b (55)	1,479	0.86	0.38	0.19	0.94	0.45	NA
Mavrogianni et al., 2019 (78)	2,116	0.83	0.82	NA	NA	NA	0.82
Nieto-Martinez et al. Men FINDRISC 5 2019a (79)	1,438	0.9	0.36	NA	NA	NA	NA
Nieto-Martinez et al. Women FINDRISC 5 2019b (79)	1,623	0.93	0.3	NA	NA	NA	NA
Nieto-Martinez et al. Men FINDRISC 6 2019c (79)	1,438	0.86	0.44	NA	NA	NA	NA
Nieto-Martinez et al. Women FINDRISC 6 2019d (79)	1,623	0.89	0.39	NA	NA	NA	NA
Nieto-Martinez et al. Men FINDRISC 7 2019e (79)	1,438	0.81	0.49	NA	NA	NA	NA
Nieto-Martinez et al. Women FINDRISC 7 2019f (79)	1,623	0.82	0.46	NA	NA	NA	NA
Nieto-Martinez et al. Men FINDRISC 8 2019g (79)	1,438	0.78	0.56	NA	NA	NA	NA
Nieto-Martinez et al. Women FINDRISC 8 2019h (79)	1,623	0.79	0.55	NA	NA	NA	NA
Nieto-Martinez et al. Men FINDRISC 9 2019i (79)	1,438	0.72	0.62	NA	NA	NA	NA
Nieto-Martinez et al. Women FINDRISC 9 2019j (79)	1,623	0.71	0.6	NA	NA	NA	NA
Nieto-Martinez et al. Men FINDRISC 10 2019k (79)	1,438	0.6	0.7	NA	NA	NA	NA
Nieto-Martinez et al. Women FINDRISC 10 2019l (79)	1,623	0.71	0.65	NA	NA	NA	NA
Nieto-Martinez et al. Men FINDRISC 11 2019m (79)	1,438	0.53	0.76	NA	NA	NA	NA
Nieto-Martinez et al. Women FINDRISC 11 2019n (79)	1,623	0.68	0.71	NA	NA	NA	NA
Nieto-Martinez et al. Men FINDRISC 12 2019o (79)	1,438	0.46	0.81	NA	NA	NA	NA
Nieto-Martinez et al. Women FINDRISC 12 2019p (79)	1,623	0.54	0.77	NA	NA	NA	NA
Nieto-Martinez et al. Men FINDRISC 13 2019q (79)	1,438	0.39	0.85	NA	NA	NA	NA
Nieto-Martinez et al. Women FINDRISC 13 2019r (79)	1,623	0.43	0.83	NA	NA	NA	NA
Rajput et al., 2019 (83)	892	0.84	0.58	0.31	0.94	0.79	NA
Abdallah et al., 2020 (80)	713	0.74	0.43	NA	NA	NA	NA
Bahijri et al., 2020 (39)	NA	0.69	0.69	0.4	0.88	NA	0.76
Ephraim et al. FINDRISC 2020a (58)	135	0.58	0.87	NA	NA	NA	0.76
Ephraim et al. MetS 2020b (58)	135	0.75	0.72	NA	NA	NA	0.74
Mao et al., 2020 (63)	7,675	0.66	0.63	NA	NA	NA	0.75
Lim et al., 2020 (65)	293	0.86	0.49	NA	NA	NA	0.76
Jamhangiry et al., 2020	440	0.99	0.53	0.81	0.95	0.84	0.84
Sengupta et al., 2021 (81)	325	0.83	0.83	0.62	0.93	NA	0.83
Abbas et al., 2021 (38)	1,454	0.86	0.58	0.5	0.9	NA	0.8
Shdaifat et al. FBG>100 Finnish 2021a (71)	392	0.45	0.93	0.79	0.75	0.76	NA
Shdaifat et al. FBG>100 British 2021b (71)	392	0.53	0.78	0.58	0.74	0.69	NA
Shdaifat et al. FBG>100 Australian 2021c (71)	392	0.9	0.49	0.5	0.9	0.64	NA
Shdaifat et al. FBG>100 Cadian 2021d (71)	392	0.25	0.79	0.4	0.65	0.59	NA
Shdaifat et al. FBG>100 German 2021e (71)	392	0.34	0.61	0.33	0.62	0.51	NA
Shdaifat et al. FBG>100 ADA 2021f	392	0.34	0.59	0.32	0.61	0.5	NA
Shdaifat et al. FBG>126 Finnish 2021g (71)	392	0.66	0.87	0.48	0.94	0.84	NA
Shdaifat et al. FBG>126 British 2021h	392	0.61	0.72	0.28	0.91	0.7	NA
Shdaifat et al. FBG>126 Australian 2021i (71)	392	0.95	0.4	0.22	0.98	0.48	NA
Shdaifat et al. FBG>126 Canadian 2021j (71)	392	0.22	0.77	0.15	0.85	0.69	NA
Shdaifat et al. FBG>126 German 2021k (71)	392	0.39	0.63	0.16	0.85	0.59	NA
Shdaifat et al. FBG>126 ADA 2021l (71)	392	0.36	0.61	0.14	0.84	0.57	NA
Shdaifat et al. PreD Finnish 2021m (71)	392	0.6	0.91	0.68	0.88	0.84	NA
Shdaifat et al. PreD British 2021n (71)	392	0.59	0.75	0.42	0.85	0.71	NA
Shdaifat et al. PreD Australian 2021o (71)	392	0.96	0.44	0.35	0.97	0.57	NA
Shdaifat et al. PreD Canadian 2021p (71)	392	0.23	0.78	0.25	0.76	0.65	NA
Shdaifat et al. PreD German 2021q (71)	392	0.33	0.61	0.21	0.74	0.54	NA
Shdaifat et al. PreD ADA 2021r (71)	392	0.33	0.59	0.2	0.74	0.53	NA
Dong et al. LR 2022a (41)	619	0.89	0.62	0.31	0.97	NA	0.81
Dong et al. ML 2022b (41)	619	0.79	0.74	0.36	0.95	NA	0.82
Dong et al. LR 2022 (41)	619	0.89	0.62	0.31	0.97	NA	0.81
Dong et al. ML 2022 (41)	619	0.79	0.74	0.36	0.95	NA	0.82
Fleming et al., 2022 (56)	406	0.94	0.23	NA	NA	NA	0.72
Han et al. XGBoost 2022a (37)	1,546,269	NA	NA	NA	NA	0.69	0.86
Han et al. RF 2022b (37)	1,546,269	NA	NA	NA	NA	0.66	0.82
Han et al. LR 2022c (37)	1,546,269	NA	NA	NA	NA	0.65	0.81
Han et al. FCN 2022d (37)	1,546,269	NA	NA	NA	NA	0.56	0.76
Henjum et al., 2022 (47)	308	0.89	0.65	0.28	0.97	NA	0.81
Jin et al., 2022 (53)	713	0.45	0.9	NA	NA	NA	0.71
Nicolaisen et al. PreDiab 2022 (35)	486,495	0.68	0.66	NA	NA	NA	0.73
Sadek et al., 2022 (50)	930	0.78	0.69	0.45	0.91	NA	0.77
Arrdóttir et al. >9 points 2023a (54)	220	0.93	0.53	NA	NA	NA	0.81
Arrdóttir et al. >10 points 2023b (54)	220	0.79	0.67	NA	NA	NA	NA
Arrdóttir et al. >11 points 2023c (54)	220	0.79	0.67	NA	NA	NA	NA
Arrdóttir et al. >12 points 2023d (54)	220	0.76	0.73	NA	NA	NA	NA
Arrdóttir et al. >13 points 2023e (54)	220	0.69	0.81	NA	NA	NA	NA
Arrdóttir et al. >14 points 2023f (54)	220	0.55	0.84	NA	NA	NA	NA
Arrdóttir et al. >15 points 2023g (54)	220	0.41	0.89	NA	NA	NA	NA
Mugume et al.>4 2023a (57)	1,764	0.73	0.57	0.04	0.99	0.65	0.75
Mugume et al.>5 2023b (57)	1,417	0.7	0.66	0.05	0.99	0.68	0.75
Mugume et al.>6 2023c (57)	1,110	0.65	0.73	0.06	0.99	0.69	0.75
Mugume et al. >7 2023d (57)	858	0.9	0.8	0.07	0.99	0.7	0.75
Mugume et al. >8 2023e (57)	638	0.56	0.85	0.09	0.99	0.7	0.75
Mugume et al. >9 2023f (57)	472	0.5	0.89	0.11	0.99	0.69	0.75
Zheng et al. Men 2023a (64)	15,665	NA	NA	NA	NA	NA	0.74
Zheng et al. Women 2023b (64)	2,719	NA	NA	NA	NA	NA	0.76
Yonel et al., 2023 (77)	3,339	0.79	0.5	0.26	0.92	NA	0.69
Mugume et al. M>4 2023 (57)	1,764	0.73	0.57	0.04	0.99	0.65	NA
Mugume et al. M>5 2023 (57)	1,417	0.7	0.66	0.05	0.99	0.68	NA
Mugume et al. M>6 2023 (57)	1,110	0.65	0.73	0.06	0.99	0.69	NA
Mugume et al. M>7 2023 (57)	858	0.6	0.8	0.07	0.99	0.7	NA
Mugume et al. M>8 2023 (57)	638	0.56	0.85	0.09	0.99	0.7	NA
Mugume et al. M>9 2023 (57)	472	0.5	0.89	0.11	0.99	0.69	NA
Mugume et al. S>4 2023 (57)	1,531	0.73	0.63	0.05	0.99	0.68	NA
Mugume et al. S>5 2023 (57)	1,219	0.68	0.71	0.06	0.99	0.69	NA
Mugume et al. S>6 2023 (57)	920	0.59	0.78	0.06	0.99	0.68	NA
Mugume et al. S>7 2023 (57)	723	0.58	0.83	0.08	0.99	0.7	NA
Mugume et al. S>8 2023 (57)	484	0.51	0.88	0.1	0.99	0.7	NA
Mugume et al. S>9 2023 (57)	396	0.43	0.91	0.11	0.99	0.67	NA
Mugume et al., 2023 (57)	4,027	NA	NA	0.08	NA	NA	0.75

#### 3.1.2 All cause cardiovascular disease

The pooled C statistic for All Cause Cardiovascular Disease was 0.77 (CI 0.71, 0.84) across a population of 42,631. When assessing the C-statistic for prediction scores of the development of all cause cardiovascular disease, the Chi^2^ heterogeneity was 8.9e+07, the I^2^ variation attributable to heterogeneity was 100% and the Tau^2^ between-study variance was 0.0055.

The pooled Hazard Ratio for All Cause Cardiovascular Disease was 1.55 CI 1.38, 1.71) across a population of 22,512. When assessing the Hazard Ratio for all cause cardiovascular disease, the Chi^2^ heterogeneity was 1.73, and the I^2^ variation attributable to heterogeneity was 0.

The pooled Risk Ratio for All Cause Cardiovascular Disease was 1.29 (CI 1.26, 1.32) across a population of 2,824,371 (**Figure 3**) and was found to have a Chi^2^ heterogeneity 98.45 and I^2^ variation attributable to heterogeneity was 93.9%.

#### 3.1.3 Hypertension

The pooled C Statistic for Hypertension was 0.77 (CI 0.77, 0.78) across a population of 20,039. When assessing the C-Statistics of prediction scores of the development of hypertension, the Chi^2^ heterogeneity was 5.2e+06, the I^2^ variation attributable to heterogeneity was 100% and the Tau^2^ estimate of between-study variance was 0.0001.

#### 3.1.4 Stroke

The pooled Risk Ratio for Stroke was 1.67 (CI 1.59, 1.76) across a population of 852,402 (**Figure 4**). When the Stroke Risk Ratio was assessed, the Chi^2^ heterogeneity was found to be 0.25 and the I^2^ variation attributable to heterogeneity was 0%.

### 3.2 Prehypertension risk of bias

Amongst the 29 study subgroups which underwent PROBAST (17) ‘risk of bias’ evaluation (**Figure 2a**), 86% (25/29) study subgroups were found to have some concerns of bias and 14% (4/29) studies were found to have low bias. In the subdomain analysis, concerns of bias were found to be 41% (12/29) in the Participants section, 10% (3/29) in the Predictors section, 28% (8/29) in the Outcome section and 17% (5/29) in the Analysis section. Any discrepancy involved a senior third colleague being consulted. Individual studies which met the inclusion criteria were included in the statistical analysis, with checks included to ensure no duplication of results under analysis.

#### 3.2.1 Prediabetes

**Figure 5:**
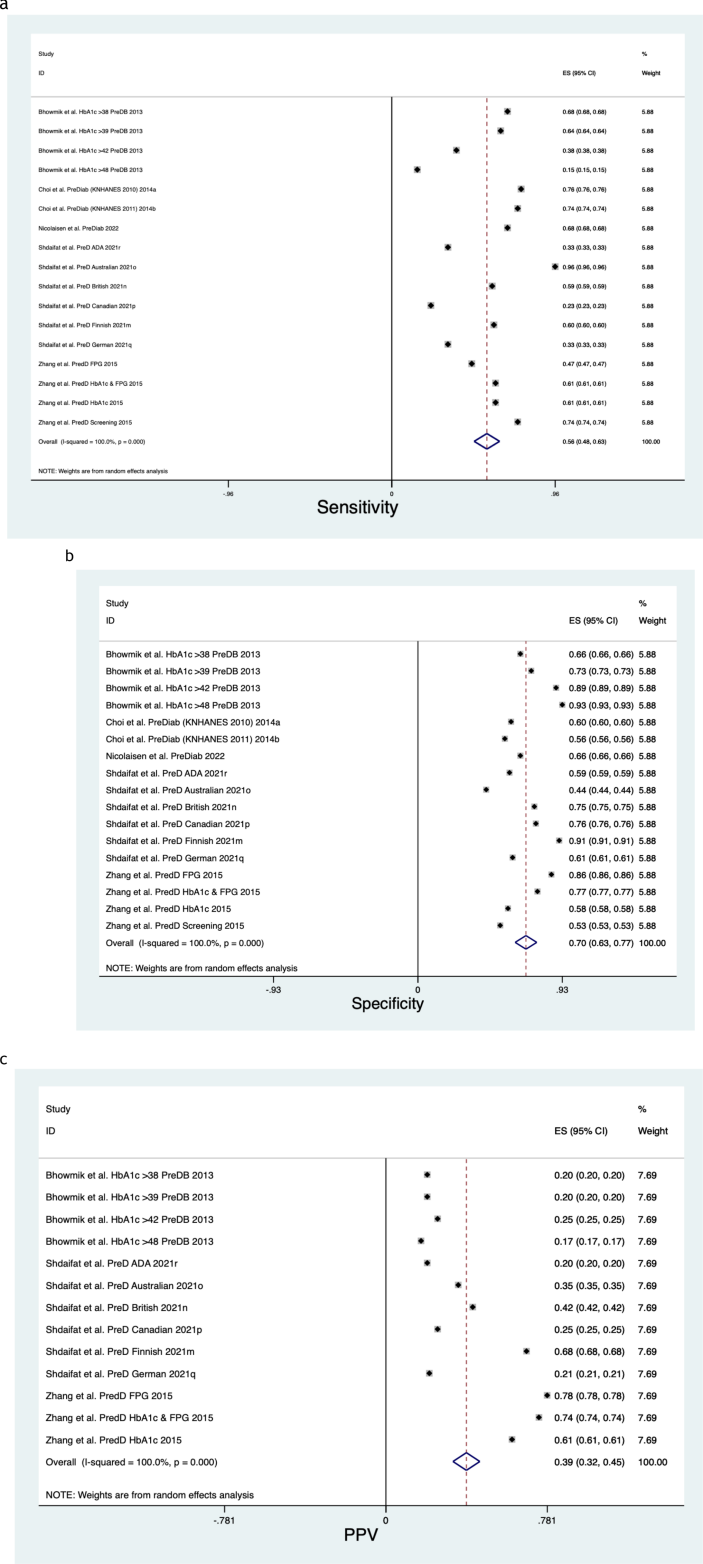
**(a)** Prediabetes sensitivity forest plot. **(b)** Prediabetes specificity forest plot. **(c)** Prediabetes positive predictive value forest plot.

**Figure 6:**
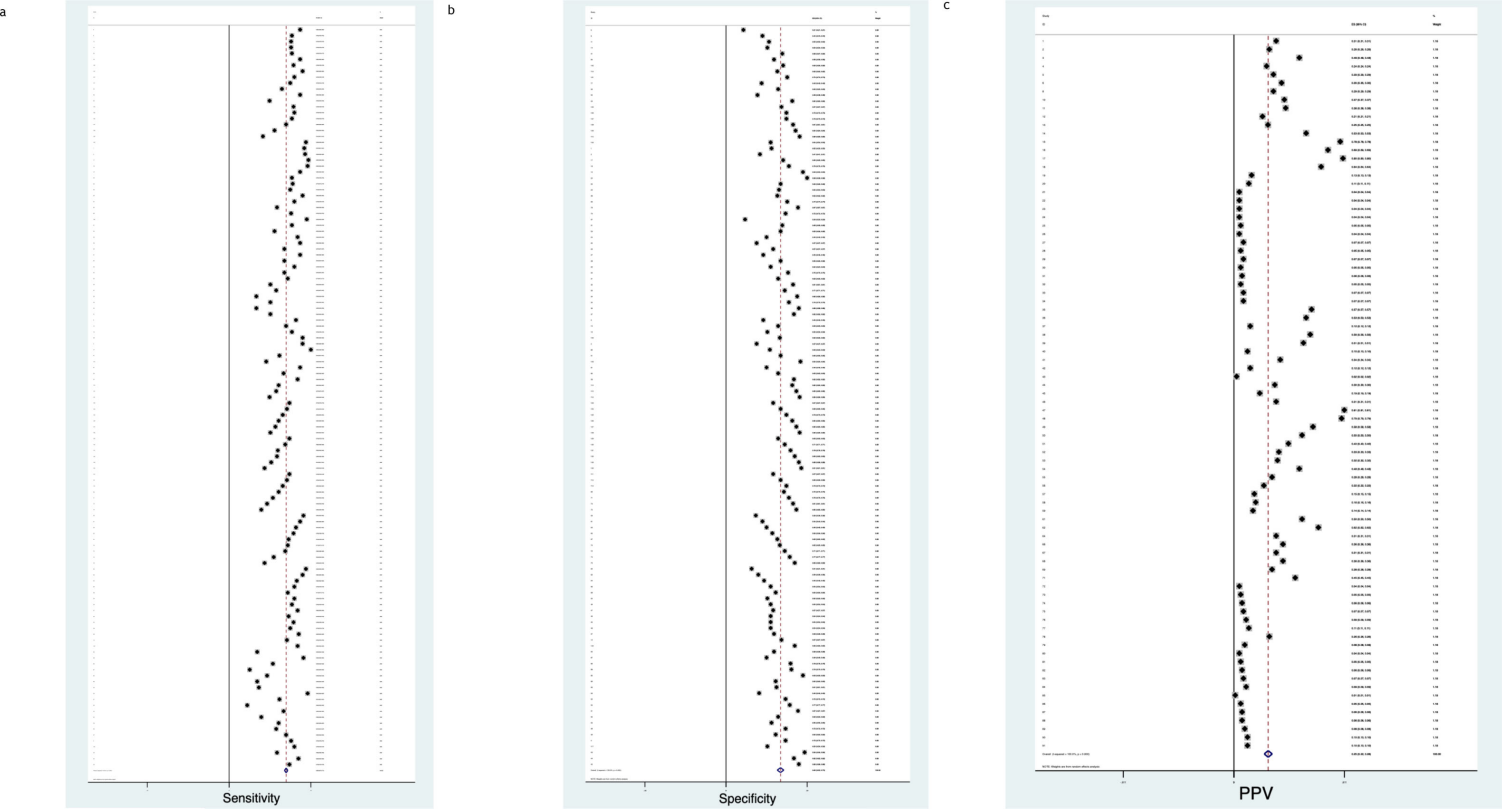
**(a)** Diabetes sensitivity forest plot. **(b)** Diabetes specificity forest plot. **(c)** Diabetes positive predictive value forest plot.

The prediabetes search identified 1500 relevant citations. After removing duplicate results, 1345 articles were screened for titles and abstracts, and 116 studies were included for full-text review. 65 articles were excluded after full-length review due to lack of predictive clarity as per the PROBAST criteria. Thus, 51 studies were eligible for inclusion in the study (**Figure 1b**), with a total of 2,193,555 patients represented in the final meta-analysis, after accounting for the risk of double counting patients in different studies. The descriptive variables are displayed in **Table 1b**. **Table 2b** provides a Summary of Results. **Figure 2b** describes the PROBAST (17) Risk of Bias assessment. **Figures 5** and **6** describe the subgroups of results.

### 3.3 All cause diabetic disease spectrum

- *Sensitivity*

When assessing All Cause Diabetic Disease Spectrum risk, the pooled Sensitivity was 0.68 (CI 0.65, 0.7), with a Chi^2^ heterogeneity 1.6e+09, an I^2^ variation attributable to heterogeneity 100% and a Tau^2^ estimate of between-study variance of 0.0156.

- *Specificity*

When assessing All Cause Diabetic Disease Spectrum risk, the pooled Specificity was 0.66 (CI 0.64, 0.67), with a Chi^2^ heterogeneity 2.2e+09, an I^2^ variation attributable to heterogeneity 100% and a Tau^2^ estimate of between-study variance of 0.0267.

- *Positive Predictive Value*

When assessing All Cause Diabetic Disease Spectrum risk, the pooled Positive Predictive Value was 0.27 (CI 0.24, 0.30), with a Chi^2^ heterogeneity 2.2e+09, an I^2^ variation attributable to heterogeneity 100% and a Tau^2^ estimate of between-study variance of 0.0193.

*Pre Diabetes*

**Figure 5** describe the meta-analysis for Prediabetes risk, representing 497,240 patients in total.

- *Sensitivity*

When assessing Prediabetes risk, the pooled Sensitivity was 0.56 (CI 0.48, 0.63) (**Figure 5a**), with a Chi^2^ heterogeneity 1.2e+08, an I^2^ variation attributable to heterogeneity 100% and a Tau^2^ estimate of between-study variance of 0.0248.

- *Specificity*

When assessing Prediabetes risk, the pooled Specificity was 0.70 (CI 0.63, 0.77) (**Figure 5b**), with a Chi^2^ heterogeneity 1.3e+08, an I^2^ variation attributable to heterogeneity 100% and a Tau^2^ estimate of between-study variance of 0.0215.

- *Positive Predictive Value*

When assessing Prediabetes risk, the pooled Positive Predictive Value was 0.39 (CI 0.32, 0.45) (**Figure 5c**), with a Chi^2^ heterogeneity 9.7e+06, an I^2^ variation attributable to heterogeneity 100% and a Tau^2^ estimate of between-study variance of 0.0143.

#### 3.3.1 Diabetes

**Figure 6** describe the meta-analysis for Diabetes risk, representing 1,696,315 patients in total.

- *Sensitivity*

When assessing Diabetes risk, the pooled Sensitivity was 0.69 (CI 0.67, 0.71) (**Figure 6a**), with a Chi^2^ heterogeneity 6.2e+08, an I^2^ variation attributable to heterogeneity 100% and a Tau^2^ estimate of between-study variance of 0.0136.

- *Specificity*

When assessing Diabetes risk, the pooled Specificity was 0.66 (CI 0.62, 0.70) (**Figure 6b**), with a Chi^2^ heterogeneity 1.7e+09, an I^2^ variation attributable to heterogeneity 100% and a Tau^2^ estimate of between-study variance of 0.0540.

- *Positive Predictive Value*

When assessing Diabetes risk, the pooled Positive Predictive Value was 0.25 (CI 0.22, 0.28) (**Figure 6c**), with a Chi^2^ heterogeneity 2.1e+09, an I I^2^ variation attributable to heterogeneity 100% and a Tau^2^ estimate of between-study variance of 0.0192.

### 3.4 Prediabetes risk of bias

Amongst the 50 study subgroups which underwent PROBAST (17) ‘risk of bias’ evaluation (**Figure 2b**), 80% (40/50) studies were found to have some concerns of bias and 20% (10/50) studies were found to have low bias. In the subdomain analysis, concerns of bias were found to be 52% (26/50) in the Participants section, 4% (2/50) in the Predictors section, 58% (29/50) in the Outcome section and 4% (2/50) in the Analysis section. Any discrepancy involved a senior third colleague being consulted. Individual studies which met the inclusion criteria were included in the statistical analysis, with checks included to ensure no duplication of results under analysis.

## 4 Discussion

We performed a dual domain systematic review to evaluate the accuracy of risk tools to predict cardiovascular morbidity in prehypertension & diabetic morbidity in prediabetes. We found that predictive performance was generally accurate. However, there remain limitations due to confounders and methodological inconsistency, such as timeframe, which undermines comparison. We found that the pooled C statistic for All Cause Cardiovascular Disease was 0.77 (CI 0.71, 0.84) and the Hazard Ratio for All Cause Cardiovascular Disease was 1.55 (CI 1.38, 1.71). When assessing All Cause Diabetic Disease Spectrum risk, the pooled Sensitivity was 0.68 (CI 0.65, 0.7) and the pooled Specificity was 0.66 (CI 0.64, 0.67).

Translation of risk modelling into health systems is challenged by population heterogeneity (85), and the reliability of reporting to enable valid comparison across specific time periods and specific endpoints. Without more consistent standards of data disclosure, academic and commercial communities may begin to polarise to serve private sector interests. However, this could be mitigated by the availability of multivariate, granular data which offers the possibility of a new ‘social contract’ (86) in which artificial intelligence serves digitally literate citizens who retain autonomy of their data. To mitigate against model drift, we need to be able to benchmark model performance using last measurement prediction (87) to facilitate comparisons of the performance from different pools of data. A ‘model-agnostic data-driven deep learning model’ (87) needs to be grounded in a physiological model to provide meaningful, explainable clinical insights. Of note is the success of the AUSDRISK tool for prediabetes screening in primary care, with a >17 score identifying 75% at risk (56). Risk scores provide valuable analysis to direct deployment of limited resources, but there are ongoing debates among health economists to define costs and deployment of preventative treatments (88). In a review of German Primary Care Diabetes and Cardiovascular Risk Scores, automated risk scores were most impactful alongside advanced information retrieval technologies (89), although patient engagement should be quantified as part of health risk in view of the role of self-management in multimorbid chronic disease (90). The optimal integration of machine learning would be the curation of the optimal variables in different populations’ risk score. This would pave the way for bespoke forecasting in ever more precise patient cohorts, with incorporation into established genetic forecasting services.

However, algorithmic fairness is an essential consideration to ensure population risk prediction tools do not exacerbate inequalities. Demographic bias is an important consideration when evaluating risks to the fairness of an algorithm. High heterogeneity and variance between studies undermines the certainty around estimates of diagnostic accuracy. The extensiveness of the heterogeneity precludes directive interpretation from the results of this analysis. Predictive models may improve over time with increased exposure to data, although the literature currently has a trend towards high-income nations, undermining the translation of applications to ‘global south’ nations who may exhibit different disease burdens and health behaviours. There are ongoing ethical concerns in the predictive modelling community regarding diversity and economics (7). It is ethically unacceptable for risk models to only serve the interests of a privileged minority of the global population.

The studies in this dual domain systematic review show substantial variation in accuracy metrics across both cardiovascular & diabetic morbidity, alongside inconsistent reporting preventing sensitivity and specificity comparisons across all studies. Most studies were challenged by inconsistent definitions of the spectrum of diabetic disease and reporting deficiencies. Confidence intervals were intermittently declared. Datasets with homogeneous groupings in specific populations, particular regions and blood glucose ranges, were especially accurate in forecasting prediabetes development. There was significant variation in the number of patients each score was assessed with, distorting the available valid comparison methods. The search strategy led us to scrutinise papers which ultimately, do not all offer what they presented. The inconsistency in predictive score performance, even the same score in different geographies, may be attributable to the context, comorbidities, diet, and recording of local patient characteristics. Predictive scores show promise in supporting clinical decision making but there is inconsistent evidence to inform regulation, best practice, and integration into ‘front line’ healthcare products.

The systematic and safe deployment of risk algorithms into clinical use requires attention paid to policy and governance, as well as technical aspects of data and deployment infrastructure. We propose nine recommendations for policymakers and commissioners, organised under an “A to I” framework.

A) Algorithmic (generalisability)

Predictive performance in these reviews was found to vary across key demographic population subgroups. The inherent differences in patient subpopulations and disease spectrum definitions threatens generalisability and subsequent plans for Personalised Electronic Health Record forecasting. Datasets with homogeneous groupings in specific populations will be especially accurate in forecasting predisease development. Ongoing challenges with heterogenous populations make local context deployment challenging. There are potential benefits to generalisability through the combination of foundation models and electronic health records: better predictive performance & sample efficiency, simple model deployment and effective engagement with multimodal data (91). However, foundation models are complex to deploy, and have unexplored safety challenges.

B) Bias

The impact of risk scores is inconsistent (92) due to bias in training data. Those patients at highest risk of developing diabetes in a time frame of five to ten years are identifiable by predictive scores (93), but the most effective method to improve disability free life expectancy and reduce complications related to metabolic disease will be through earlier intervention at the predisease end of the spectrum. This will not be realised without commercial and academic collaboration in adherence to consistent reporting standards and representative data.

C) Change and quality

A serious challenge to risk scores is performance degradation: once a risk model is deployed, there are a diminishing number of ground truths in the present day for valid comparison, and outcome data that does get collected may be contaminated by the intervention, which presents challenges to retrain the model once drift ensues (94). The new UK federated data platform may enable secure, regional data analytics with greater flexibility for local services (95), however, the new Secure Data Environments may not widen information or population diversity (95).

D) Data source

Relative to fragmented data architecture, ‘data lakes’ (96) enable more reliable training of predictive scores and more consistent reporting patterns in global collaboration on preemptive medicine. Biomarkers are important in the risk stratification for early detection (97), with a notable success of risks scores including the Polygenic Risk Score to predict susceptibility to coronary heart disease and atrial fibrillation, enabling appropriate impact through intervention and lifestyle change (98).

E) Ethics

Evidence from real-world cases (99) needs to be compiled to ensure quality training optimises diagnostic and triage accuracy (100). Further development of transparency and diversity reporting standards, such as the ‘Health sheet’ initiative (101, 102), can help reduce established ethnic inequalities in AI datasets, as per STANDING Together (103). Economic concerns remain in conversations about the potential for insurance systems to discriminate against individuals and families based on their perceived risk profiles.

F) Functionality & ‘explainability’

The expression of disease risk across predisease spectra will be helpful to stratify patients based on their Personal Health Record data. For example, an artificial intelligence for prediction could perform using a scale for hypo- and hyper-glycemia risk, as opposed to arbitrary categories, reflecting the reality of the spectrum of disease (87). The risk profile must be grounded in physiological reality relative to potential deterioration to be useful; we need to be able to explain the disease spectrum to inform intervention.

G) Governance

Leaders with training in computer and medical science are needed to direct EHR predictive modelling technologies. This emphasis on risk scores is economically justified since cardiovascular disease (CVD) risk modelling has been projected to save £68 billion, gain 4.9 million QALYs and prevent 3.4 million CVD cases over 25 years in England (104). This leadership role will require the oversight of new guidelines like STARD-AI and CONSORT-AI (105, 106), to encompass EHR risk scores which use primary care demographics and prescription history, as already applied in Victoria, Australia (107).

H) Humans in the loop

‘Humans In The Loop’ (HITL) are a safety mechanism where experts will review and modify the decision-informing outputs of an algorithmic system. The NHS needs set apart Clinical Informaticians to supervise risk scores in EHRs against multimorbidity, one of the greatest challenges facing modern health services (108). This is especially urgent whilst the burden of CVD in the young is growing (109), and the polygenic risk score only marginally improves coronary heart disease forecasting in young adults (110). HITL clinical specialty pathways will help optimise the deployment of risk scores.

I) Interoperability

Any new risk score capability will need to integrate into legacy technology in health systems. This review found that the importance of subcomponents of a risk score differed according to the population. Set apart Clinical Informaticians are especially important to supervise the application of risk scores which otherwise systematically underestimate risk in particular ethnic, socioeconomic and chronic disease groups (111). False negatives are best mitigated with disease catalogues for underprivileged groups to improve the integration of risk score software into clinical practice (89).

## 5 Limitations

### 5.1 Prehypertension

Bias resulted from retrospective studies in which documentation, symptoms and follow up outcomes will vary across geographies. Variance in performance is hard to account for in a cross-sectional study, although there may be improvement in predictive reliability as input data grows in fidelity and volume to characterise forecasted prognosis more accurately. Analysis was undertaken on hypertension diagnosis, stroke, and all cause cardiovascular disease, however, the definitions of these events differed in reporting. The review itself was limited by the short search strategy, despite many duplications showing comprehensive coverage of the relevant material.

### 5.2 Prediabetes

Studies rarely engaged in external validation and often struggled to demonstrate that the target population was representative. Those scores focusing on prediabetes had a lack of transparency about the cut-off points for defining prediabetes and there was significant variation in the metrics of performance. The studies lacked a reliable method of demonstrating predictive accuracy and did not conduct reports transparently. The review itself was limited by the short search strategy, despite many duplications showing comprehensive coverage of the relevant material.

## 5 Conclusion

In this systematic review, cardiovascular & diabetic risk tool accuracy prediction varied due to reporting standards but was most valuable in all cause cardiovascular mortality as a useful warning system which could be deployed to an EHR national screening programme. The risk tools are consistent and valuable in predicting hypertensive risk, but there are ongoing concerns about unrepresentative training data. Artificial intelligence may have a role in the curation of variables to build the optimal algorithm for different populations, deployed as an Application Programming Interface in EHRs. However, governance decisions are challenging due to model drift and bias. Further work is needed to characterise the specific time points along the spectrum of cardiovascular & diabetic disease which signify acceleration in clinical deterioration, enabling accurate forecasting.

## Data Availability

The original contributions presented in the study are included in the article/supplementary material. Further inquiries can be directed to the corresponding author.
